# SEOM clinical guidelines for endometrial cancer (2017)

**DOI:** 10.1007/s12094-017-1809-9

**Published:** 2017-12-13

**Authors:** A. Santaballa, X. Matías-Guiu, A. Redondo, N. Carballo, M. Gil, C. Gómez, M. Gorostidi, M. Gutierrez, A. Gónzalez-Martín

**Affiliations:** 1 Servicio de Oncología Médica, Hospital Universitari i Politècnic La Fe, Valencia, Spain; 2 Servicio de Anatomía Patológica, Hospital Universitario de Bellvitge, Barcelona, Spain; 3 Servicio de Oncología Médica, Hospital Universitario La Paz, Madrid, Spain; 4 Servicio de Oncología Radioterápica, MD Anderson Cancer Center, Madrid, Spain; 5 Servicio de Oncología Médica, Hospital Durán i Reynals, Barcelona, Spain; 6 Servicio de Oncología Médica, Hospital Universitario Infanta Sofía, Madrid, Spain; 7 Servicio de Ginecología, Hospital Universitario de Donostia, Donostia, Spain; 8 Servicio de Oncología Médica, Hospital Unversitario de Araba, Vitoria, Spain; 9 Servicio de Oncología Médica, Clínica, Universitaria de Navarra, Madrid, Spain

**Keywords:** Endometrial cancer, Adjuvant treatment, Chemotherapy, Radiotherapy

## Abstract

Endometrial cancer (EC) is the most common gynecological cancer in developed countries. Most patients are diagnosed at an early stage with a low risk of relapse. However, there is a group of patients with a high risk of relapse and poor prognosis. Despite the recent publication of randomized trials, the adjuvant treatment of high-risk EC is still to be defined and there are many open questions about the best approach and the right timing. Unfortunately, the survival of metastatic or recurrent EC is short, due to the poor results of chemotherapy and the lack of a second line of treatment. Advances in the knowledge of the molecular abnormalities in EC have permitted the development of promising targeted therapies.

## Methodology

These guidelines have been developed by a multidisciplinary team of experts from Spanish Ovarian Cancer Research Group (GEICO) and SEOM for gynecological malignancies. First, each section was written by an expert and then all the authors discussed the results and determined the level of evidence and the grade of recommendation, according to the Infectious Diseases Society of America-US Public Health Service Grading System. The final text was reviewed and approved by all the authors. The goal of this document consists of providing clear practical recommendations about the management of endometrial cancer (EC).

## Introduction

EC is the most common gynecological cancer in developed countries. Although most EC patients have a favorable prognosis, those either with high-risk early disease or advanced stage at diagnosis have a survival below 50%.

The most important risk factors identified in EC are obesity, long-lasting endogenous or exogenous hyperestrogenism (polycystic ovary, tamoxifen therapy, anovulation, and nulliparity), hypertension and diabetes mellitus. In addition, women with Lynch syndrome (LS, or hereditary nonpolyposis colon cancer) are at a markedly increased risk of EC.

## Diagnosis

Abnormal uterine bleeding is the most frequent symptom of EC. Particular suspicion should be held for postmenopausal women or for those over 40 years with high-risk factors.

Transvaginal ultrasound (TVUS) is considered the first-line imaging technique to be performed. In postmenopausal women, TVUS evaluation of the endometrial thickness has a high accuracy for EC diagnosis using a cut-off value of 3 mm [II, B]. When an endometrial thickness is identified, an endometrial biopsy should be performed. Endometrial sampling is the gold standard for histologic diagnosis. If bleeding persists or recurs after endometrial sampling with benign findings, hysteroscopy should be performed.

The role of preoperative studies is to establish risk groups and to define the surgical management. A chest radiograph should be performed as a part of the initial assessment. Contrast-enhanced MRI is the best method for detecting myometrial invasion or cervical involvement, when compared with non-enhanced MRI, ultrasound, or CT scan. MRI is also the best imaging modality, compared with CT or positron emission tomography (PET) with or without CT, for detecting lymph node metastases. There is no evidence for the clinical usefulness of CA 125 in the pretreatment evaluation of EC [IV, B].

## Hereditary endometrial cancer

The majority of ECs are associated with sporadic mutations. The genetic mutations associated with LS are responsible for 2–5% of all the ECs, mainly affected by the MLH1 and MSH2 genes. The estimated lifetime risk of EC varies between 18% in MLH1 and 30% in MSH2 mutation carriers [1]. The Amsterdam II criteria and revised Bethesda Guidelines can be used to identify women with LS. The incidence of EC was significantly lower in the largest retrospective cohort study in women with the germline MLH1, MSH2 or MSH6 mutation, who underwent risk-reducing surgery versus those who did not [2].

Molecular screening for LS should be performed in women with Amsterdam or Bethesda criteria and women with EC before 50 years [II, A]. Prophylactic surgery with hysterectomy and bilateral salpingo-oophorectomy should be offered in women with LS mutations who have completed childbearing [IV, B].

## Screening

There are no high-quality data to support the efficacy of screening for reducing EC mortality.

Routine screening of asymptomatic women at average or increased risk of EC is not recommended [II, A]. In women with LS, screening should be offered in asymptomatic women who have not completed childbearing or women that refused prophylactic surgery beginning at the age of 30–35 or 5–10 years prior the earliest age of first diagnosis of Lynch-associated cancer in the family. Annual endometrial sampling [II, A], TVUS with endometrial aspiration and serum CA 125 are usually recommended to be performed every year [IV, B].

## Pathology and molecular biology of endometrial carcinoma

WHO’s classification of EC defines seven different types of tumor: endometrioid carcinoma (~ 80% of EC), usual type and variants, mucinous adenocarcinoma (1–9% of EC), serous carcinoma (< 10% of EC), clear cell carcinoma (< 5% of EC), neuroendocrine carcinoma, mixed carcinoma and undifferentiated and dedifferentiated carcinoma [3].

*Endometrioid adenocarcinoma (EEC)* and their variants are the prototype of type I EC. The usual type EEC encompasses a spectrum of neoplasms with variable histological differentiation that ranges from well-differentiated tumors (grades 1 and 2) to solid and poorly differentiated carcinomas (grade 3). EEC with squamous differentiation accounts for 25–50% of all EEC.

*Serous carcinoma* (*SC*) is the prototype of type II EC. SC is a very aggressive tumor, which arises occasionally in endometrial polyps. SC is usually associated with deep myometrial and extensive lymphovascular invasion. Tumor cells are usually positive for p53, p16, IMP2, IMP3.

*Clear Cell Carcinoma* (*CCC*) shows clear cells and a combination of patterns such as solid, papillary, glandular, and tubulocystic. HNF-1 beta, AMACR and Napsin A immunostaining are usually expressed.

SC or CCC may coexist with EEC. When more than one of these components is present at least in 5% of the tumor, it is diagnosed as a *mixed carcinoma*.

The molecular genetic alterations of EEC (type I) differ from those of SC (type II), and cDNA analysis shows different gene expression profiles. Whereas EEC shows microsatellite instability (MI) and mutations in the PTEN, PIK3CA, K-RAS and CTNNB1 genes, SC have alterations of p53, chromosomal instability, as well as other molecular alterations (STK15, p16, E-cadherin, and C-erbB2) [4] (Table 1).

**Table 1:** Molecular features of endometrioid and serous endometrial cancer

Biomarker	Alteration	Frequency in EEC%	Frequency in SC%
PTEN	Loss of function	80	0–10
K-RAS	Mutation	25	4
CTNNB4/β-catenin	Mutation/nuclear expression	40	0–5
MI	Microsatellite instability	20–45	0–5
PIK3CA	Mutation	50	40
ER, PR	Expression	70–73	20–24
ARID1A	Mutation/loss of function	35	7
Stathmin	Overexpression	15	64
HER 2	Overexpression	3–10	32
E-cadherin	Loss of function	5–50	60–90
P16	Loss of function	8	45
P53	Mutation	11	80–90

*EEC* Endometrioid endometrial cancer, *SC* Serous carcinoma

The Cancer Genome Atlas Research Network (TCGA) has recently performed an integrating genomic characterization of EC [5]. Interestingly, exome sequence analysis revealed four groups of ECs: Group 1, with EEC with mutations in POLE, and showing high mutation rates (ultramutated), associated with good prognosis; Group 2, including EEC with microsatellite instability (hypermutated), and Group 3, tumors including EEC with low copy number alterations, showing similar progression-free survival rates. Group 4 (serous-like) including SC, but also EEC (usually grade 3), exhibited p53 mutations, and worse prognosis. Results show that there is a group of EEC, that are molecularly and prognostically similar to SC. Combining POLE mutational analysis with immunohistochemical analysis of p53 and mismatch repair markers (PMS-2 and MSH-6) have been proposed to classify the tumors in the four TCGA groups, particularly for high-grade EEC and SC, as a surrogate approach to apply TCGA to clinical practice. However, validation is needed.

## Staging and risk assessment

EC is surgically staged. The staging is based on FIGO 2009 (Table 2) [6]. The most important prognostic factors identified in EC are: FIGO stage, histological subtype, grade, depth of myometrial invasion, lymphovascular space invasion (LVSI), and age [7]. According to the risk of relapse, EC has been subdivided into four risk categories (Table 3). In the near future, the molecular advances could be used for outcome prediction and may aid in optimal distinction of the risk groups.

**Table 2:** FIGO classification 2009

Stage	Definition
IA	Tumor confined to the uterus, no or < ½ myometrial invasion
IB	Tumor confined to the uterus, > ½ myometrial invasion
II	Cervical stromal invasion, but not beyond the uterus
IIIA	Tumor invades serosa or adnexa
IIIB	Vaginal and/or parametrial involvement
IIIC1	Pelvic node involvement
IIIC2	Para-aortic involvement
IVA	Tumor invades bladder and/or bowel mucosa
IVB	Distant metastases including abdominal metastases and/or inguinal lymph nodes

**Table 3:** ESMO risk groups to guide adjuvant therapy use

Risk group	Description
Low risk	Stage I endometrioid G1–2, < 50% myometrial invasion, LVSI negative
Intermediate risk	Stage I endometrioid, G1–2, ≥ 50% myometrial invasion, LVSI negative
High–intermediate risk	Stage I endometrioid, G3, < 50% myometrial invasion regardless of LVSI
	Stage I G1–2, LVSI positive, regardless of depth of invasion
High risk	Stage I EEC, G3, ≥ 50% myometrial invasion, regardless of LVSI
	Stage II EEC
	Stage III EEC optimally debulked
	Non-endometrioid EC (serous or clear cell or undifferentiated carcinoma, or carcinosarcoma)

*LVSI* lymphovascular invasion, *EEC* endometrioid endometrial cancer, *EC* endometrial cancer

## Surgical treatment

### Early stages

All patients with newly diagnosed disease should be considered for surgery.

The standard surgical approach of endometrioid EC in early stages is total hysterectomy without vaginal cuff with bilateral salpingo-oophorectomy and with or without lymphadenectomy. Peritoneal cytology, although recommended is not mandatory for FIGO staging.

Lymphadenectomy (LND) provides prognostic information. Two randomized controlled trials have shown no overall survival (OS) benefit from LND in early-stage EC [8, 9]. Decisions about whether to perform lymphadenectomy and to what extent can be made based on the preoperative findings or based on the intraoperative study of the hysterectomy. Criteria indicative of low risk for nodal metastases are less than 50% of myometrial invasion, tumor less than 2 cm or grade 1 and 2.

Sentinel lymph node mapping (SLNM) provides important information to tailor adjuvant therapy and reduces LND-related morbidity and long-term sequelae of unnecessary adjuvant treatments, although published results are single-institution series or multi-institutional collaborations, without a prospective randomized trial.

Laparoscopic approach has a lower rate of surgical complications and similar outcomes than laparotomy. Robotic approach could be an alternative to laparoscopic approach, with less estimated blood loss and outcomes comparable to laparoscopy.

In non-endometrioid early stages, due to their high propensity to disseminate in the upper abdomen, complete staging including abdominal cavity review, bilateral salpingo-oophorectomy, pelvic lymphadenectomy, para-aortic lymphadenectomy up to the renal vein, omentectomy and peritoneal biopsies with maximal surgical debulking is recommended.

The standard surgical approach of endometrioid EC in early stages is laparoscopic [I, A] with total hysterectomy, without vaginal cuff and bilateral salpingo-oophorectomy [IA]. In low-risk EC, LND is not recommended [II, A]. In intermediate and high-risk group, LND is recommended to guide surgical staging and adjuvant therapy [II, C]. SLNM in EC is promising and being performed in many centers, but still can not be recommended as standard treatment. In non-endometrioid early stages, complete staging and maximal surgical debulking is recommended [IV, A].

### Advanced stages

A complete staging with maximal surgical debulking is recommended in patients with good performance status and resectable tumor [III, B]. Palliative surgery could be considered in patients with good performance status and metastasic disease [IV, A].

### Fertility sparing therapy

Reproductive age women with low-risk EC should be advised about fertility sparing options. To recommend fertility preservation, it is important to exclude evidence of myometrial invasion, extrauterine disease or non-endometrioid histologies. Progestin therapy (medroxyprogesterone acetate; 400–600 mg/day or megestrol acetate; 160–320 mg/day or an intrauterine device containing levonorgestrel) is the only available option in these patients, but there are no studies that compare progestin therapy vs standard therapy. Close follow-up and confirmation of lesion regression are mandatory.

Fertility preservation could be offered in reproductive age patients with low-risk EC, but there is no standard option [V, A]. Progestins are the recommended treatment [IV, B].

## Adjuvant treatment

Adjuvant treatment for patients with early-stage disease is tailored according to the risk group and the most important prognostic factors.

### Adjuvant radiotherapy

Pelvic radiation (PRT) after surgery in stage I EC provides locoregional control, but there is no improvement in OS or disease-free survival (DFS) [10]. A randomized trial comparing vaginal brachytherapy (VBT) and observation in women with stage IA, grade 1 and 2 endometrioid EC have shown no OS benefit in VBT group and VBT was associated with an increase in genitourinary symptoms [11]. The results of PORTEC-2 trial, that compared VBT and PRT [12] in the high–intermediate-risk group, showed that there were no differences in pelvic or vaginal recurrences, DFS and distant metastasis, but the VBT group suffered significantly lesser toxicities than the PRT group. VBT in combination with PRT was compared to VBT only in patients with intermediate risk in a randomized trial [13]. Addition of PRT improved locoregional control, but had no impact on OS and was associated with increased acute gastrointestinal and urinary toxicity. Postoperative RT has been considered standard in high-risk EC group, although a comparative study of adjuvant radiation versus no treatment in this group of patients has not been conducted.

### Adjuvant chemotherapy

Results of prospective randomized trials comparing PRT to chemotherapy (CT) in high-risk EC have shown that CT reduced the risk of distant recurrences, but did not improve OS and the local control was poor. These observations have provided the rationale for a combined CT/RT approach. The role of adjuvant combined treatment with PRT and CT in EC has been studied in patients with intermediate and high risk. The pooled analysis of NSGO-EC-9501/EORTC-55991 and MaNGO ILIADE-III trials demonstrate that combined treatment (four cycles of platinum-based CT, given either before or after RT) improve DFS and show a trend towards improved OS [14]. The limitation of these studies is that 25–40% of the patient population was stage III or incompletely surgically staged. The type of CT used and the number of cycles are other concerns that preclude generalization of these results.

The recent results of the two randomized trials have shown no benefit for adjuvant CT over PRT in high–intermediate and high-risk EC. In PORTEC-3 trial, PRT was compared with chemoradiation (two cycles of cisplatin with PRT, followed by four cycles of carboplatin and paclitaxel), with no difference in OS or DFS, but adverse events were more frequent with CT/RT. However, in stage III, there was an improvement in DFS with CT/RT with no improvement in OS [15]. In GOG 249, CT (carboplatin-paclitaxel) plus VBT have demonstrated similar DFS as PRT, but more acute toxicity [16]. The results of GOG 258, a randomized trial that compared CT vs CT-RT in stages III to IVA, I–II serous or clear cell EC have shown no differences in DFS [17]. In PORTEC-3 and GOG 258, completion rate of CT was lower after radiation and most recurrences were distant. For these reasons, in stage III EC, we recommend adjuvant CT followed by PRT, rather than the combination. The results of protocol ENGOT-EN2-DCGC will definitively address the benefit of chemotherapy in high-risk early stages.

**Fig. 1:**
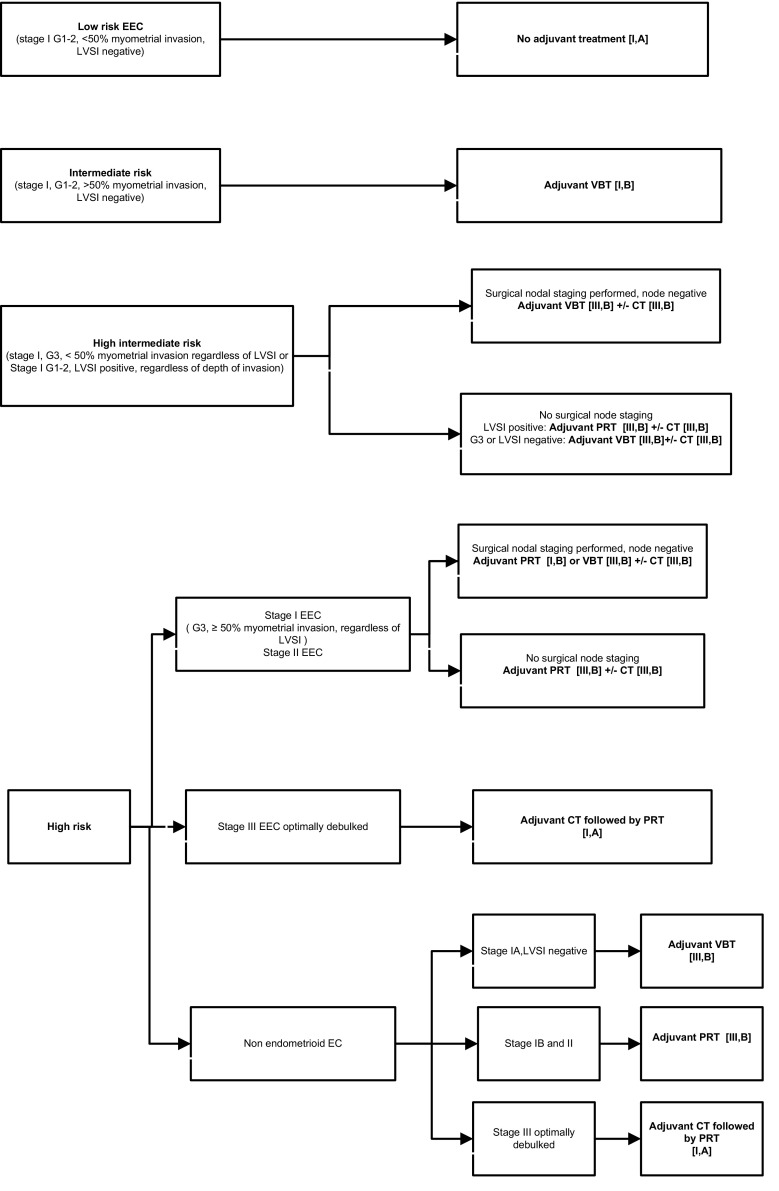
Adjuvant treatment for endometrial cancer. *LVSI* lymphovascular invasion, *VBT* vaginal brachytherapy, *CT* chemotherapy, *PRT* pelvic radiotherapy, *EEC* endometrioid endometrial cancer, *EC* endometrial cancer. Modified from endometrial cancer algorithms that refers to Colombo et al. [7]

Adjuvant treatment recommendations with the level of evidence and grade of recommendations are summarized in Fig. 1.

## Treatment of metastatic and advanced disease

Surgery or radiation (in non-irradiated area) are options in patients with isolated centropelvic recurrence or single metastasic site [IV, A]. In patients with extrapelvic relapse, CT or hormonal therapy (HT) are palliative options.

### Hormonal treatment

HT could be an option in endometrioid EC. The response rate (RR) with progestagens in first line is ~ 25%, while the RR described with tamoxifen or aromatase inhibitors is ~ 10%. Predictors of response are low histological grade, prolonged time to relapse, the location and extent of extrapelvic disease and positive hormone receptor. Hormone receptor status should be determined before initiating hormonal therapy, although its role as a predictor of response to hormonal treatment has not been clearly demonstrated [18]. The treatment of choice is a progestational agent (megestrol acetate 160 mg QD or medroxyprogesterone acetate 200 mg QD) [III, A].

Endocrine therapy is recommended as a therapeutic alternative for those patients with G1–2 tumors, hormone receptor positive and no rapid progressive disease [IV, A].

### Chemotherapy

The most active drugs in EC are doxorubicin, platinum agents and paclitaxel. Doxorubicin was compared with doxorubicin plus cisplatin (AP) in FIGO III and IV EC in two large randomized trials. The combination arm showed better RR and PFS, but no benefit in OS. Doxorubicin plus paclitaxel combination was compared to AP, and no significant differences were found in RR, DFS or OS. AP combination was compared with doxorubicin, cisplatin and paclitaxel (TAP) in advanced EC, with an improvement in RR, PFS and OS that was shown in the TAP arm, but with bad tolerability [19]. The GOG 209 trial compared carboplatin–paclitaxel (CT) versus TAP. There were no differences in OS or DFS (in the CT arm: PFS 12–14 m, OS 32 m). The better toxicity profile of CT regimen made it the standard scheme for advanced disease, but also for adjuvant treatment [20].

Available options after the first-line therapy are limited. The evaluated drugs are paclitaxel (RR 20%), liposomal doxorubicin (RR 9.5%), ifosfamide (RR 15%), oxaliplatin (RR 13. %) and ixabepilone (RR 12%).

Carboplatin and paclitaxel is the standard option in metastatic or advanced endometrial cancer [I, A]. There is no standard CT for second line.

### Targeted therapies

The identification of molecular abnormalities in EC, as described previously, has permitted the development of new drugs as target therapies. Antiangiogenic drugs and mTOR/PI3K inhibitors are the most promising agents. EGFR and Her-2 neu inhibitors have had disappointing results. Up to now, no approved targeted therapies are available for EC.

#### Antiangiogenic drugs

Bevacizumab has been evaluated as a monotherapy agent in phase II trials, resulting in a RR of 15.1%, PFS 4.2 and OS 10.5 months and, in combination with paclitaxel and carboplatin with a RR of 73%. Latest phase II randomized trials (GOG86P and MITO END-2) have shown an interesting activity in combination with carboplatin and paclitaxel in first line, in advanced or metastatic EC [21, 22]. Phase III trials with bevacizumab combination are ongoing.

#### mTOR inhibitors

Several phase II trials have been reported with mTOR inhibitors. The largest randomized study observed a PFS of 3.6 months for the experimental arm versus 1.9 months for the hormonotherapy treatment group. These drugs have shown low RR (0–25%), but the clinical benefit is secondary to long disease stabilization. Preclinical data have suggested that mTOR inhibition reverses hormonal resistance. A phase II trial with letrozole and everolimus has been reported with 32% of RR.

## Follow-up

Most EC relapses occur within 3 years after primary treatment. The pelvis is the most common site of recurrence, most of them in the vaginal vault, whereas distant relapses account for only one-third of all the cases. Most patients with EC will have a low risk of recurrence and more than one-half of all the recurrences will be detected with examination and symptoms. Recurrent EC has poor prognosis, regardless of the time of detection, with the exception of local relapse.

There are no prospective studies that have evaluated the role of the surveillance in EC. The most consistently used method is the physical examination which includes a thorough speculum, pelvic, and rectovaginal examination. Cytology does not add any clinical benefit [23]. The CA-125 level should not be used routinely in patients with EC, but may be appropriate in selected patients with advanced disease, serous histology or a higher CA-125 level at diagnosis [24]. The routine use of chest RX is not recommended. Pelvic US and CT may play a role in the evaluation of patients with symptoms, advanced stages or clinical signs of recurrence.

All recommendations for the diagnosis, treatment and follow-up of EC are summarized in Table 4.

**Table 4:** SEOM guidelines recommendations for the management of endometrial cancer

Diagnosis
TVUS and endometrial sampling should be considered the standard approach [II, B]
Women with EC should have contrast-enhanced MRI and chest RX before surgery [IV, A]
Hereditary endometrial guidelines
Molecular screening for LS should be performed in women with Amsterdam or Bethesda criteria and women with EC before 50 years [II, A]. Prophylactic surgery with hysterectomy and bilateral salpingo-oophorectomy should be offered in women with LS mutations who have completed childbearing [IV, B]
Screening
Routine screening of asymptomatic women at average or increased risk of endometrial carcinoma is not recommended [II, A]
Staging
EC is surgically staged. The staging is based on FIGO 2009 [IV, A]
Surgical treatment
The standard surgical approach of endometrioid EC in early stages is laparoscopic [I, A] with total hysterectomy without vaginal cuff and bilateral salpingo-oophorectomy [IA]. In low-risk EC LND is not recommended [II,A]. In intermediate and high-risk group, LND is recommended to guide surgical staging and adjuvant therapy [II, C]. SLNM in EC is still not recommended as standard treatment. In non-endometrioid early stages, complete staging and maximal surgical debulking is recommended [IV, A]
In advanced stages, a complete staging with maximal surgical debulking is recommended in patients with good performance status and resectable tumor [III, B]. Palliative surgery could be considered in patients with good performance status and metastasic disease [IV, A]
Fertility preservation could be offered in reproductive age patients with low-risk EC, but there is no the standard option [V, A]. Progestins are the recommended treatment [IV, B]
Adjuvant treatment
Low-risk patients do not require adjuvant treatment [I, A]
VBT is recommended for intermediate-risk patients [I, A]
In the intermediate–high-risk group, VBT is recommended in patients with surgical staging and node negative [III, B]. CT can be evaluated [III, B]. In patients with no surgical nodal staging, PRT and VBT is recommended [III, B]
In high-risk early stages, endometrioid EC, VBT [III, B] or PRT [III, B] are recommended. In early stages with non-endometrioid histologies VBT [IIIB] or PRT [I, B],CT can be evaluated [III, B]. The recommendation in stage III optimally debulked is CT, followed by PRT [I, B]
Treatment of advanced or recurrent disease
Surgical or local treatment (radiation in non-irradiated area) are options in patients with isolated centropelvic recurrence or single metastasic site [IV, A]
Endocrine therapy is recommended as a therapeutic alternative for those patients with well-differentiated tumors or a long disease-free interval [IV, A]
Carboplatin and paclitaxel is the standard option in metastatic or advanced endometrial cancer [I, A]. There is no standard CT for second line
Follow-up
Physical examination with a thorough speculum, pelvic, and rectovaginal examination is the most effective method for the detection of EC recurrences [IV, A]
Cytology evaluation and chest RX are not recommended in asymptomatic women, imaging test should be reserved for patients with suspected recurrence [IV, A]
